# Dynamic global tracker for online multi camera multi vehicle tracking

**DOI:** 10.1038/s41598-026-35768-z

**Published:** 2026-01-24

**Authors:** Xiaoxiang Chen, Sixian Chan, Guo Bin, Yuan Yao, Feng Hong, Jiafa Mao, Xiaolong Zhou

**Affiliations:** 1 College of Computer Science and Technology, Zhejiang University of Technology, Hangzhou, 310023 China; 2 Hubei Key Laboratory of Intelligent Vision Based Monitoring for Hydroelectric Engineering, College of Computer and Information, China Three Gorges University, Yichang, 443002 China; 3 Hangzhou GANX Science & Technology Co., Ltd, Hangzhou, 310051 China; 4 School of Computer Science, University of Nottingham Ningbo, Ningbo, 315100 China; 5 College of Information Science and Technology, Zhejiang Shuren University, Hangzhou, 310000 China; 6 College of Electrical and Information Engineering, Quzhou University, Quzhou, 324000 China

**Keywords:** MCMT, Online tracking, Vehicle ReID, Multi-video analysis, Mathematics and computing, Computer science

## Abstract

Multi-camera multi-target Tracking (MCMT) is often regarded as a downstream task of Multi-Object Tracking (MOT). Traditional methods typically follow an offline pipeline involving detection, re-identification, single-camera tracking, and post-hoc clustering, which leads to poor real-time performance, high computational cost, and weak adaptability in dynamic environments. Moreover, trackers tailored for specific locations overly rely on manually crafted information like road topology and camera calibration, reducing their effectiveness in varied scenarios. We propose Dynamic Global Tracking (DGT), an innovative online framework for Multi-Camera Multi-Target (MCMT) vehicle tracking. Unlike traditional methods that rely on full trajectory extraction and then clustering, the DGT integrates cross-camera associations directly into the tracking process. This transformation reduces the computational burden and enhances real-time performance. Especially, our framework includes a Hybrid Fusion Module (HFM) to address resolution disparities and a Stable Trajectory Manager (STM) to improve stability and robustness. Extensive experiments demonstrate that DGT significantly improves tracking accuracy and adaptability in various environments, achieving an IDF1 score of 61.19 on the HST dataset (speed version) and 70.49 (performance version) with FPS of 90.

## Introduction

With the rise of autonomous driving^1,2^ and smart cities^3^, intelligent transportation systems (ITS) play an increasingly vital role in enhancing traffic efficiency and safety. ITS^4,5^ continuously monitors real-time traffic flow and detects abnormal behaviors, providing essential support for traffic management and decision-making. A key component of ITS is Multi-Camera Multi-Target Tracking (MCMT), which enables multiple cameras to operate collaboratively, covering larger areas and offering more comprehensive traffic information. Unlike single-camera tracking(SCT), MCMT emphasizes inter-camera association, addressing complex factors such as camera position, orientation, and overlapping fields of view. In real-world MCMT vehicle tracking, challenges such as congestion, traffic accidents, and irrelevant obstacles add significant complexity to road conditions.

**Fig. 1:**
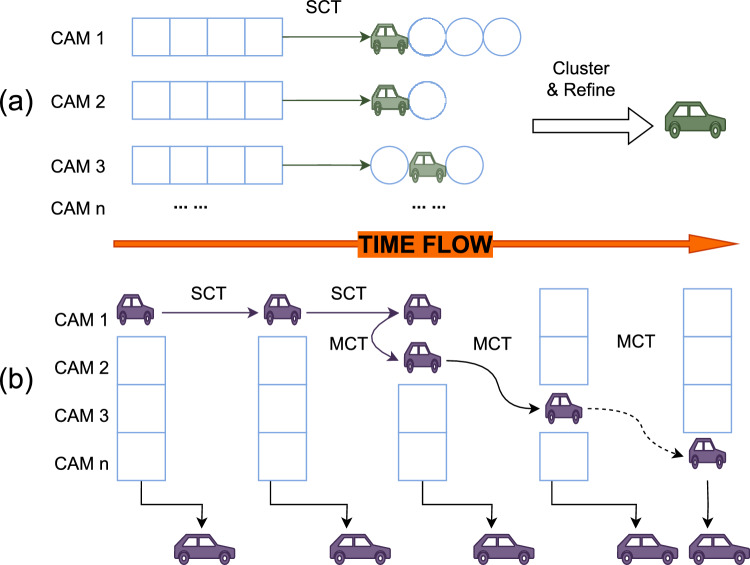
(**a**) Offline: The offline method in MCMT is retrospective, clustering and refining past data for comprehensive post-event analysis. (**b**) Online: The online approach provides immediate, real-time tracking and track prediction for dynamic response in multi-camera systems, where SCT is short for single camera tracking and MCT is short for multi-camera tracking.

In MCMT tasks, traditional methods typically involve object detection, single-camera tracking, and trajectory clustering. Some approaches utilize more refined detectors^6–8^ and appearance feature extractors^9–13^ to obtain location and appearance embedding vectors of target vehicles. Subsequently, various MOT methods^14–18^ are applied to refine partial trajectories within individual camera views, termed as local trajectories. Ultimately, clustering techniques^19–21^ are used to discern tracks with similar trajectory characteristics, thereby achieving cross-camera association. These methods are known as off-line methods, as shown in the first mode of Fig. 1.

Methods based on this scheme leverage road topology^8^ and camera calibration^12^ for cross-view target association, reducing clustering overhead. Nonetheless, as traffic environments grow increasingly intricate, current techniques encounter multiple hurdles in real-world scenarios. Firstly, the computational complexity of these methods remains high due to the need to cluster and match a large number of trajectories, making it difficult to achieve real-time processing. Secondly, reliance on manually crafted information increases the complexity of deployment and maintenance. Thirdly, the accumulation of large volumes of historical data exacerbates the challenges of real-time processing, further limiting the practical applicability of these approaches. Lastly, existing methods often fail to address the discrepancies in resolution and perspective between different cameras, leading to suboptimal tracking accuracy.

This paper aims to develop an efficient, real-time MCMT framework capable of achieving high-precision vehicle tracking in complex traffic environments. The specific contributions are as follows:

1. **DGT**: Based on an iterative matching strategy, the framework integrates cross-camera associations into the tracking process, significantly improving real-time performance and accuracy.
2. **HFM**: Using a dual-strategy approach to integrate multi-scale features, effectively addressing resolution and perspective differences between different cameras.
3. **STM**: To meet the needs of cross-view tracking, the system’s trajectory representation and management are redesigned, enhancing adaptability and robustness.

## Related work

Traditional MCMT system encompasses four main processes: detecting and locating vehicles in each camera’s view, distinguishing vehicle appearances for re-identification, generating raw tracks in individual cameras using semantic and motion information, and finally, linking these tracks across cameras to form complete tracks through clustering and post-processing.

### Vehicle semantic feature extractor

Vehicle Re-Identification (Re-ID) aims to recognize the same vehicle across cameras, relying on a structured feature embedding vector to handle challenges from varied camera angles, lighting conditions, and environmental changes [**9650856**]. In the context of MCMT, both in- and inter-camera recognition heavily depend on the cosine similarity of appearance features. Using an ensemble of various backbone networks is a common approach^22^. This ensemble helps networks complement each other, enhancing details and leading to a notable increase in training and inference time.

Besides, re-ranking techniques like k-reciprocal encoding^23^ are often employed in MCMT to improve the relevance of search results by reordering the initial search outcomes. This technique is widely used in image retrieval, video search, and recommendation systems^24,25^. Although re-ranking improves the accuracy of tracking and identification, it also introduces additional computational complexity, which may impact the ability to achieve real-time results.

### Multi object tracking

The MOT task aims to track multiple targets within a single camera view and is a crucial foundation for the MCMT. SORT (Simple Online and Real-time Tracking)^26^ enhances the detector performance by using the Kalman filter^27^ for prediction. Subsequently, it employs the classical Hungarian matching in frame association. On this basis, Deep SORT^9,28^ proposes the fusion of location information and deep appearance features for matching to enhance the stability of the tracker. Additionally, Deep SORT introduces Cascade Match, prioritizing targets based on the interval since the last successful matching. Targets with more frequent matches are given priority, thus improving tracking accuracy.

Transformer-based models have recently achieved strong results in single-camera multi-object tracking (MOT) by modeling long-range dependencies and improving robustness against occlusions. Frameworks such as TransTrack integrate detection and tracking into a unified network, using attention mechanisms to associate objects across frames. These models also dynamically combine motion and appearance features, avoiding hand-crafted matching rules.

However, applying Transformers to multi-camera multi-object tracking (MCMT) presents several challenges. First, their quadratic computational complexity becomes a major bottleneck when dealing with multiple video streams. Second, large cross-camera appearance differences and lighting variations can degrade the effectiveness of attention-based matching. Finally, the high real-time demands of MCMT systems make heavy Transformer architectures impractical in many scenarios. Therefore, more lightweight and modular approaches—such as two-stage tracking pipelines—remain better suited for MCMT applications at this stage.

### Multi camera association

Partial tracks within the camera are obtained after detection, semantic feature extraction, and single camera tracking (SCT). This stage aims to associate these tracks through clustering, widely adopted in most studies, *e.g.*, fast clustering^29^, cross-camera trajectory matching^30^, and hierarchical clustering^31^. However, these methods typically rely on analyzing complete video data, leading to low efficiency. To alleviate these issues, some work tries to reduce the number of cluster candidates based on spatio-temporal information, such as road direction^32,33^ or adjacent temporal fragments^34,35^. Additionally, world coordinates obtained through camera calibration^36^ and high-dimensional appearance embeddings^22^ are leveraged to enhance accuracy. However, these methods do not fundamentally address the significant burden of clustering. Furthermore, cluster-based methods fail to meet the demands of real-time application scenarios since they require complete video data as input. Moreover, manually designed information for intersection*s, such as entry or exit zones, is labor-intensive and difficult to transfer.

Consequently, some research^37^ explores tracking over time by maintaining a global track pool. Another approach^34^ clusters data at the current moment but associates it temporally, and results are outputted in time delay, thereby breaking the “offline batch processing” model. However, these methods can cause ambiguous target identification with camera overlapping and require additional computation. Our proposed DGT approaches this differently by separately processing each camera and employing binary graph matching instead of clustering. As a result, this approach is highly versatile and applicable to various scenarios, working effectively regardless of camera overlapping.

## Methods

### System architecture

**Fig. 2:**
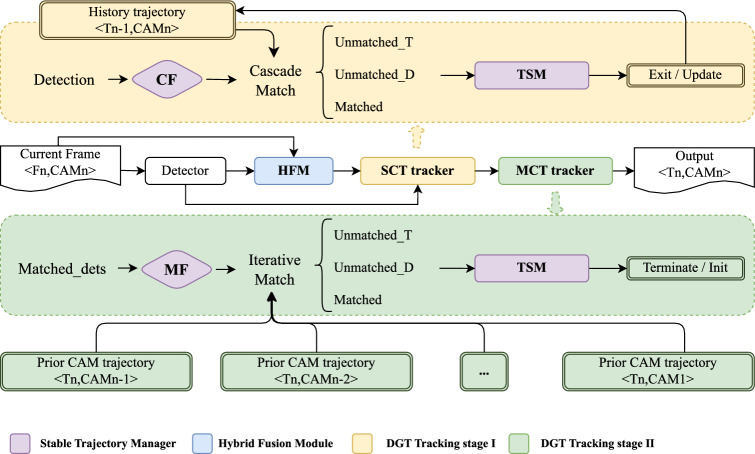
Flowchart of the proposed online framework DGT. **Detector**: Any vehicle detector generating bounding boxes with confidence scores. **HFM**: Our proposed Hybrid Fusion Module extracts deeply fused multi-scale appearance features. **SCT tracker**: Generates partial tracks through Cascade Match and assigns local IDs. **MCT tracker**: Connects vehicles across cameras using Iterative Match and assigns global IDs. **CF**: Confidence Filter, a threshold for the detector-generated confidence score. **TSM**: Temporal Stability Mechanism determines whether a trajectory should be updated or deactivated (when it exits the view), terminated, or initiated. **MF**: Movement Filter, minimal displacement of matched targets, with movements smaller than this value considered as obstructions or non-target entities.

Figure 2 illustrates the architecture of the proposed online framework. The system consists of several key components: object detection and feature extraction using the HFM in the DGT, which includes the SCT stage through Cascade Matching and the MCT stage through Iterative Matching. The object detector generates bounding boxes with confidence scores for detected vehicles. These are then processed by the HFM to extract robust scale-invariant features. SCT generates local trajectories, which are passed to the MCT stage for cross-camera association using the Hungarian algorithm.

We redesigned the trajectory representation. Each trajectory records the target’s historical bounding boxes, appearance features, and other basic information within the camera’s view, maintaining a dynamically updated ID list. The DGT framework carefully manages global IDs to maintain consistency and continuity in target identification across various camera views. Unlike traditional MCMT methods, SCT and MCT processes occur simultaneously between frames in DGT. This integrated strategy culminates in the generation of real-time decision-support reports, essential for applications requiring rigorous urban safety and traffic management.

Besides, to ensure the accuracy and adaptability of the DGT, we designed the Stable Trajectory Manager (STM), in which several essential components work together to aid in tracking:

- Confidence Filter (CF): This component ensures the quality of the detection results by filtering out low-confidence detections, thereby improving the reliability of the tracked targets.
- Temporal Stability Mechanism (TSM): This mechanism uses time thresholds to determine when a target has entered or exited the camera’s view, handling temporary disappearances due to occlusion or scene changes. It provides reasonable initialization and termination of targets, ensuring robust and continuous tracking.
- Movement Filter (MF): This component removes outputs with no displacement in track matching for an extended period, ensuring that non-target obstacles do not interfere with the tracking process.

By synergizing all the above, the system optimizes resource distribution and significantly enhances tracking continuity and overall system performance. The detailed workflow of the system’s main modules will be elaborated in the following section*s.

### Hybrid fusion module

The Hybrid Fusion Module is designed to robustly integrate features across multiple scales through a dual-strategy approach. This innovative module processes input images using a feature pyramid, capturing a comprehensive range of details from fine to coarse scales, feeding these feature maps into two distinct fusion strategies.

**Fig. 3:**
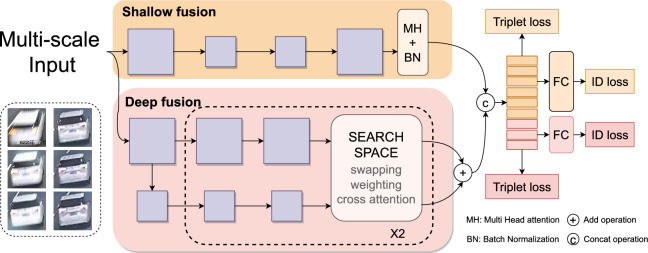
The model structure of our proposed Hybrid Fusion Module. Features are extracted parallelly using a classical re-id backbone for shallow fusion and a deep fusion model derived from Neural Architecture Search (NAS). For the deep fusion model, we introduce swapping, weighting, and cross-attention to construct a potential multi-scale interaction search space.

As shown in Fig. 3, The Shallow Fusion Strategy employs Multi-Head (MH) attention and Batch Normalization (BN) to merge features while preserving their original detail and texture. This technique is crucial for quickly capturing fine-grained visual information and is especially effective in scenarios where high-resolution detail is paramount. Conversely, the Deep Fusion Strategy utilizes a Neural Architecture Search (NAS) optimized network to abstract and consolidate broader contextual cues. It incorporates advanced techniques such as swapping weights and cross-attention within its search space, enhancing the processing of multi-scale feature maps for more comprehensive integration.

After processing the features through both the shallow and deep fusion paths, the outputs of these two branches are concatenated. This integration capitalizes on the strengths of both strategies: the shallow fusion contributes detailed, immediate recognition capabilities, while the deep fusion adds depth with its contextually rich, abstracted features. The synergy of these outputs is then finely tuned using triplet loss $\mathcal {L}_{t r i}$ (Eq. 1)to ensure tight clustering of similar objects across various scales and feature classification loss $\mathcal {L}_{id}$ (Eq. 2) to maintain high discriminative power for precise identification.

1 $$\begin{aligned} & \mathcal {L}_{t r i}=\left[ \mathcal {D}\left( \textbf{f}_{a}, \textbf{f}_{p}\right) -\mathcal {D}\left( \textbf{f}_{a}, \textbf{f}_{n}\right) +m\right] _{+} \end{aligned}$$

where, $\textbf{f}_{a}$, $\textbf{f}_{p }$, $\textbf{f}_{n }$ are the embedded features for the anchor, the hardest positive and negative samples in a mini-batch, $\mathcal {D}(\cdot , \cdot )$ is the Euclidean distance, m is the margin parameter, and $\left[ \right] _{+}$ is the $max(\cdot , 0)$ function, and

2 $$\begin{aligned} & \mathcal {L}_{id}=\frac{1}{N} \sum _{i=1}^{N}-\log \left( \frac{\exp \textbf{W}_{i}^{\top } \textbf{f}_{i}}{\sum _{j} \exp \textbf{W}_{j}^{\top } \textbf{f}_{i}}\right) & \end{aligned}$$

where, $\textbf{f}_{i }$ is a feature vector, the classifier weight is $\textbf{W}_{i }$.

This streamlined methodology ensures that the Hybrid Fusion Module significantly enhances object recognition capabilities under various conditions, making it a powerful tool for complex visual tasks.

### Dynamic global tracking

In the DGT system, the iterative matching strategy with global ID assignment is utilized to achieve efficient multi-camera target tracking.

**Algorithm 1:**
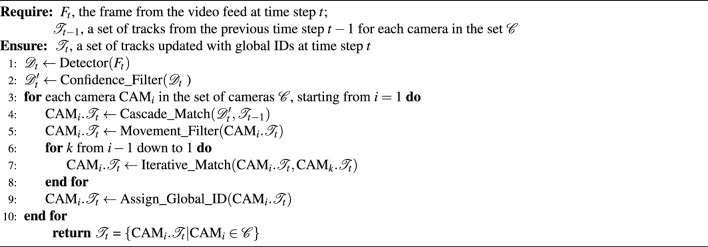
Iterative matching strategy.

During the SCT stage, each camera independently detects and tracks targets, using cosine distance between deep features($C_{dist}$) fused with pixel distance between bounding boxes($P_{dist}$) as the cost matrix, formulated in Eq. 3 and Eq. 4. This matrix helps determine whether they belong to the same vehicle ID based on appearance similarity and positional distance. During this stage, we acquire the local tracks currently being monitored by each camera, complete initialization or Kalman updates for active trajectories, and assign a local ID to each one after cascade matching^15^.

3 $$\begin{aligned} & C_{dist}(A, B)=1-\frac{A \cdot B}{\Vert A\Vert \Vert B\Vert } \end{aligned}$$

4 $$\begin{aligned} & P_{dist}(A,B)=\frac{1}{4} \sum _{i\in \left\{ t,l,b,r \right\} }\sqrt{A_{i}^{2}-B_{i}^{2} } & \end{aligned}$$

where, *A* and *B* mean two different detected targets or trajectories. *t*, *l*, *b*, and *r* donate the top, left, bottom, and right corners, respectively.

these newly updated or initialized local trajectories, without needing to be fully formed, are immediately passed to the multi-camera tracking (MCT) stage. In this stage, we utilize an iterative matching strategy: the Hungarian algorithm is employed to sequentially match these trajectories from other cameras. Successfully matched trajectories are assigned or updated with a global ID, while unmatched trajectories remain in a matching pool for subsequent rounds. Each successful match is recorded in the dynamic ID list. After processing all frames at the current time step, the records of each trajectory are analyzed, and the ID with the highest frequency in history is selected as the final target’s global ID. The specific process can be referred to in the Algorithm 1.

Assume that within a given time window, *n* vehicles pass through *m* non-overlapping cameras. Traditional clustering-based approaches typically require at least $O(m^{2}n^{2})$ time complexity due to exhaustive pairwise comparisons across all cameras. In contrast, our method distributes approximately *n*/*m* active vehicles per camera and performs matching in a distributed manner. As a result, the worst-case time complexity for cross-camera association is reduced to $O(n^{2})$, significantly lowering the overall computational cost.

Additionally, in TSM, different time thresholds are used for the SCT and MCT stages to ensure real-time accuracy of trajectories in both local and global perspectives. This Iterative Matching and dynamic adjustment mechanism allows adaptation in real-time to target movements and environmental changes, ensuring consistent target identification and tracking across multiple camera views. This optimizes resource utilization and reduces computational load, providing efficient and reliable target tracking

## Experiments

We conducted experiments on both the Highway Surveillance Traffic (HST) dataset and the AI City Challenge, *e.g. *, the CityFlowV2 dataset^38^.

**Fig. 4:**
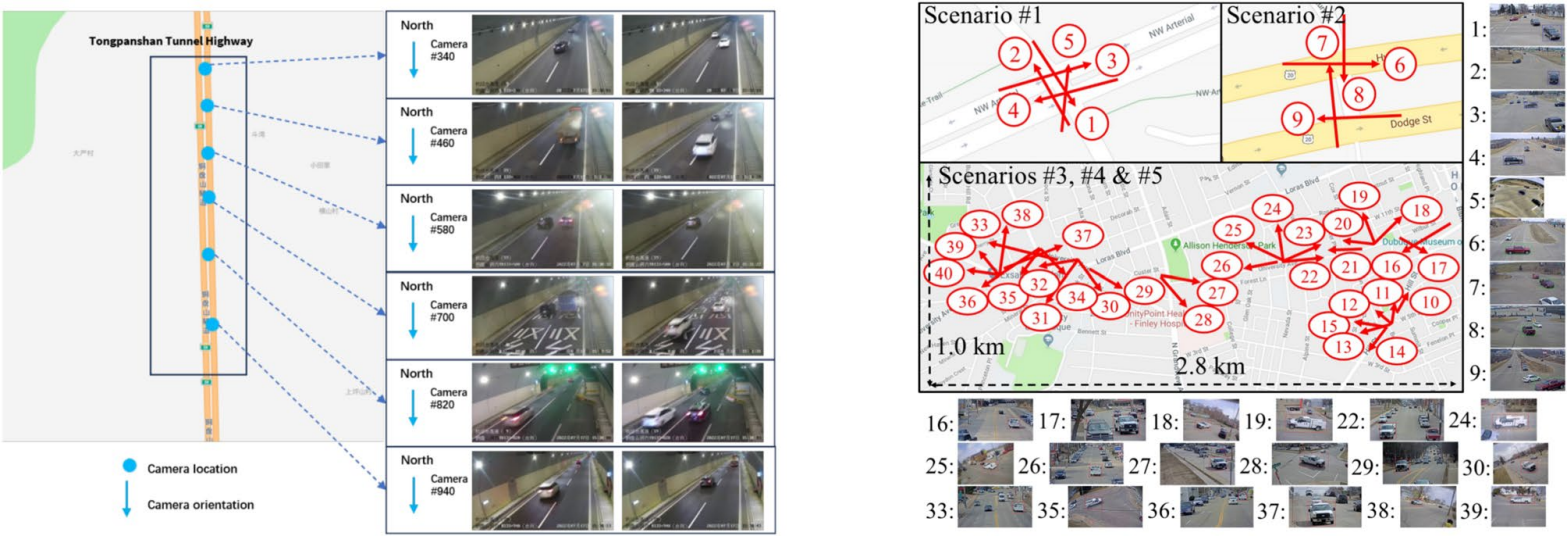
(**a**): Illsutration of the HST Dataset information; (**b**): Illsutration of the CityFlow Dataset information

### Datasets & evaluation

HST: Our team established the Highway Surveillance Traffic (HST) dataset by meticulously gathering data from the Tongpanshan Tunnel section* of the Hangzhou-Shaoxing-Taizhou Highway in China. The data were collected using six cameras (C340, C460, C580, C700, C820, C940) positioned consecutively along the Tongpanshan Tunnel, covering the north-to-south direction. The videos captured by these six cameras have a resolution of 1920 × 1080 pixels, and the dataset spans a total duration of 3. 03 hours (182 minutes). The HST dataset includes a diverse range of commonly occurring vehicle types, such as cars, trucks, buses, and more.

The AI City Challenge: The dataset for Challenge Track 1, the CityFlowV2^38^ dataset, is designed explicitly for Multi-Camera Multi-Target (MCMT) tracking and is provided by NVIDIA. It comprises video data collected from 16 cameras situated in a mid-sized city in the United States, covering a total duration of 3.58 hours (215.03 minutes). Notably, the subset S2 serves as our test set. Unlike the linear road scenes in the HST dataset, S2 documents an intersection* scenario with significant camera overlap, as shown in the Fig. 4.

To assess the performance of the MTMCT system, this paper utilized the IDF1 metric. IDF1 measures the ratio of correctly identified detections to the ground truth and also considers the average number of calculated detections. Meanwhile, the IDP, IDR, and MOTA metrics were employed. The specific definitions of IDF1, IDP, IDR, and MOTA are given as follows, where IDSW represents the count of identifier errors caused by identifier switches, while GT represents the count of detections in the ground-truth tracking set:

5 $$\begin{aligned} \begin{aligned}I D P&=\frac{I D T P}{I D T P+I D F P} \\I D R&=\frac{I D T P}{I D T P+I D F N} \\I D F 1&=\frac{2 I D T P}{2 I D T P+I D F P+I D F N} \\M O T A&=1-\frac{F N+F P+I D S W}{G T}\end{aligned} \end{aligned}$$

### Comparison with state-of-the-art

**Table 1:** State-of-the-arts comparison on HST and AICity Challenge S02. *indicates that the results of MCMT tracking are reproduced by us, using the same detector and appearance feature. The best results are in **bold**.

Method	Dataset	Online	IDF1 $\uparrow$	IDP $\uparrow$	IDR $\uparrow$	MOTA $\uparrow$	FPS $\uparrow$
CityScale*^32^	HST		**78. 34**	**80.54**	**76. 26**	**70.50**	18
Dyglip*^39^	HST		66. 75	73. 69	61. 01	55. 75	5
Electricity*^40^	HST		56. 23	73. 24	45. 63	35. 16	15
WCMSM^41^	HST		73.67	81.09	67.50	58.38	-
DSRF^42^	HST		77.17	81.89	72.96	58.42	-
ASTM-Net^8^	HST		79.84	78.72	81.01	62.71	-
LW-Detector^43^	HST		78.02	77.65	79.42	60.32	-
OURS	HST	$\checkmark$	70.51	80.36	62. 83	57. 36	**90**
CityScale*^32^	S02		56. 72	42. 10	**86. 91**	-13. 49	12
NCCU^36^	S02		45. 97	48. 91	43. 35	-	-
UMD_RC^44^	S02		44. 13	63. 57	33. 80	-	-
Ocaware_MCT^45^	S02		48. 17	34. 82	78. 12	-	-
EffinetDet_MCT^34^	S02		64. 26	55. 15	76. 98	-	-
Dyglip*^39^	S02		**64. 90**	**67. 05**	70.43	**63. 29**	3
Online_MCT^37^	S02	$\checkmark$	40.60	39. 70	41. 60	-	-
OURS	S02	$\checkmark$	56. 48	56. 84	56. 22	35. 57	**58**

Specifically, for the detection stage, we have enhanced the YOLOv5^46^ by incorporating the window attention mechanism from Swin Transformer^47^, achieving an AP of 95.08% at IoU 0.5 and 91.10% at 0.75. A minor modification of enlarging detection results and introducing background information has proven beneficial for extracting more semantic information. Additionally, during the feature extraction phase, we conducted comparative experiments using both the highest accuracy performance version and the fastest balanced version.

We compare our proposed Multi-Camera Multi-Target tracker with the state-of-the-art in both camera overlapping scenes (AICity Challenge S02) and non-overlapping scenes (HST). To facilitate a better comparison of tracker performance, we utilize the same detection results and appearance feature embeddings during the reproduction process. Due to a lack of camera information in the HST reproduction, we omit the module involving homogeneous coordinates. It is evident that our approach is the fastest. In addition, we also report several representative HST results from recent competitive pipelines (e.g., WCMSM, DSRF, ASTM-Net, and LW-Detector) in Table 1. These methods achieve strong accuracy on the non-overlapping HST benchmark, indicating that further gains are still possible when the pipeline is allowed to adopt heavier detectors, stronger feature extractors, or more computation-intensive association strategies. However, these approaches typically do not target real-time online tracking, and their runtime (FPS) is often not explicitly reported or is substantially lower in practice. In contrast, our tracker is designed as an online method and maintains high efficiency (90 FPS on HST and 58 FPS on S02), achieving a better balance between robustness and deployability for practical multi-camera systems.

Moreover, regardless of whether camera perspectives overlap, our method proves effectiveness without additional design, while methods designed for specific scenes may fail in others. For instance, as shown in Table 1, CityScale^32^, the winner of the AICity Challenge 2021, performs exceptionally well in HST but experiences a significant MOTA collapse (-13.49) in AICity S02. DyGLIP^39^achieves an IDF1 of 64.90 on AICity S02 but performs less favorably than our method on HST (66.75 vs. 70.51). Overall, our online approach demonstrates outstanding robustness and reliability across diverse scenes.

### Experiment of the HFM

**Table 2:** Comparison of HFM across various backbones. “Ensemble” refers to averaging the features outputted by ResNet101, ResNext101, and Se_Resnet101.

Backbones	HFM	IDF1 $\uparrow$	IDP $\uparrow$	IDR $\uparrow$	MOTA $\uparrow$	**Params** $\downarrow$	**FLOPs** $\downarrow$
Resnet101		44. 88	52. 34	40.04	56. 53	44. 8M	29.27G
Resnext101		46. 32	54. 26	41. 33	56. 32	44. 4M	29.39G
Se_Resnet101		24. 84	28. 27	22. 15	56. 73	49. 5M	29.30G
Resnet50		59. 04	67. 77	52. 56	57. 03	24. 0M	12.08G
Ensemble		70.49	80.34	62. 81	57. 36	138.0M	/
MSINet		54. 03	62. 91	48. 71	56. 45	12.5M	5.14G
Resnext101	$\checkmark$	52. 27	61. 37	46. 61	56. 74	46. 6M	/
Renet50	$\checkmark$	61. 19	70.08	54. 54	57. 15	26. 4M	/
Ensemble	$\checkmark$	70.51	80.36	62. 83	57. 36	140.0M	/

In this section, we conduct experiments on 3 NVIDIA RTX A6000 GPUs. Various backbones for our Hybrid Fusion Module are trained on the Veri^48^, VehicleID^49^, and AIC_ReID datasets^50^, using triplet loss and ID loss as penalty terms. The results are shown in the Table 2, where the ensemble is obtained by averaging the 2048-dimensional features outputted by ResNet101^9^, ResNext101^10^, and Se_Resnet101^51^ backbones.

**Fig. 5:**
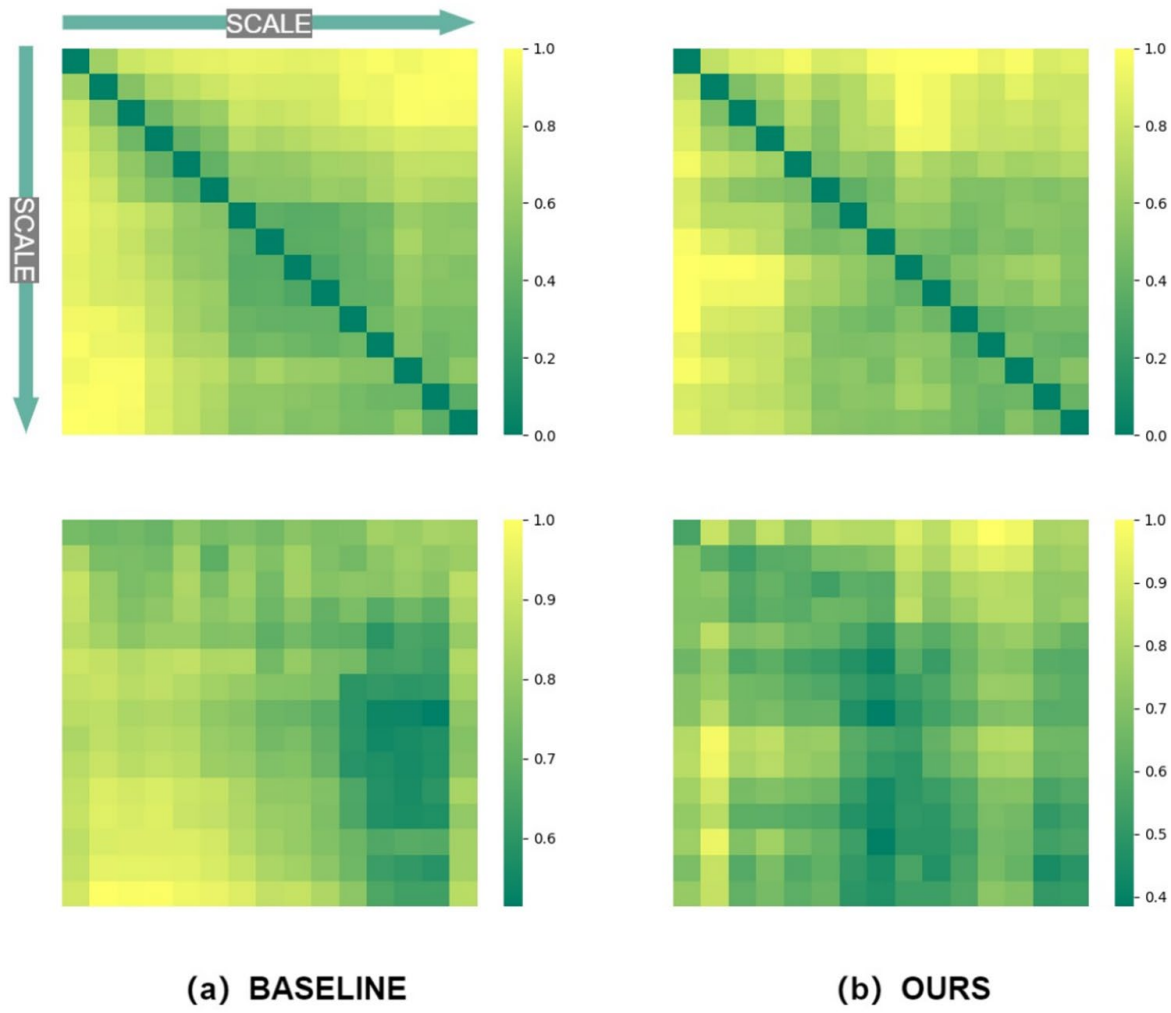
Baseline vs. Ours: Visualization of appearance feature distance at different scales for the same target. We standardized the data to the range [0,1]. The first row illustrates the comparison between the baseline method and our approach in in-camera scenes, showing that our approach significantly reduces the variance in appearance feature distances, leading to more stable tracking. The second row indicates scenarios involving inter-camera settings, where our method again demonstrates reduced variance and improved feature consistency. This enhanced consistency across scales and cameras is crucial for accurate cross-camera association and tracking.

Table 2 presents the performance of the Hybrid Fusion Module (HFM) integrated with different backbone networks. Notably, MSINet, as the deep fusion branch alone, achieves competitive results with the smallest parameter count. Combining a simple ResNet50 network with the deep fusion branch can achieve an IDF1 of 61.19, striking a good balance between speed and accuracy. In our performance version, combining the ensemble with our deep fusion branch can achieve an IDF1 of 70.51, demonstrating competitive performance compared to offline methods.

Figure 5 demonstrates the impact of resolution changes on target re-identification and the effectiveness of the Hybrid Fusion Module (HFM) in addressing this issue. We selected images of the **same** target captured at different times and from different cameras, showing variations in scale due to distance changes. The experiment compared the baseline model with the improved model incorporating HFM (Ours) by extracting features from these images and generating feature similarity matrices, which are visualized using heatmaps.

As Fig. 5 shown, In the baseline model, features of the similar scale showed higher correlation, as indicated by the high values along the diagonal of the heatmap. However, the correlation significantly decreased when the scale varied, leading to lower accuracy in re-identification. In the model with HFM (Ours), the feature similarity remained high even with significant scale differences, resulting in a more uniform distribution of high values in the heatmap. This indicates that HFM provides more stable feature extraction and integration across different resolutions, improving the robustness and accuracy of vehicle re-identification. In summary, HFM significantly enhances the model’s ability to handle varying resolutions and scales in target re-identification, ensuring more stable and effective feature extraction and fusion.

### Ablation study of the STM

To ensure the accuracy and adaptability of the Dynamic Graph Tracker (DGT), we designed several essential components in the Stable Trajectory Manager(STM). We conducted ablation experiments on them to demonstrate their effectiveness, as shown in Table 3. The Confidence Filter (CF) ensures the quality of the detection results, while the strategy design for Temporal Stability Mechanism (TSM) provides the physical logic of target movement. This allows vehicles entering and leaving cameras to have a reasonable initialization and termination, addressing the temporary disappearance of targets due to occlusion or scene changes. Finally, outputs with no displacement in track matching for an extended period shall be removed in the Matching Filter (MF), ensuring immunity to interference from non-target obstacles in the field.

**Table 3:** Ablation for Components in STM, where CF, TSM and MF represent the confidence filter, temporal stability mechanism and movement filter, respectively.

Iterative Match	CF	TSM	MF	IDF1 $\uparrow$	IDP $\uparrow$	IDR $\uparrow$	MOTA $\uparrow$
$\checkmark$				47. 22	70.19	35. 57	31. 66
$\checkmark$	$\checkmark$			51. 97	58. 99	47. 26	60.13
$\checkmark$	$\checkmark$	$\checkmark$		60.25	68. 64	54. 78	60.56
$\checkmark$	$\checkmark$		$\checkmark$	58. 57	64. 54	34. 28	56. 13
$\checkmark$		$\checkmark$	$\checkmark$	51. 48	52. 62	52. 08	46. 59
$\checkmark$	$\checkmark$	$\checkmark$	$\checkmark$	70.49	80.34	62. 81	57. 36

**Fig. 6:**
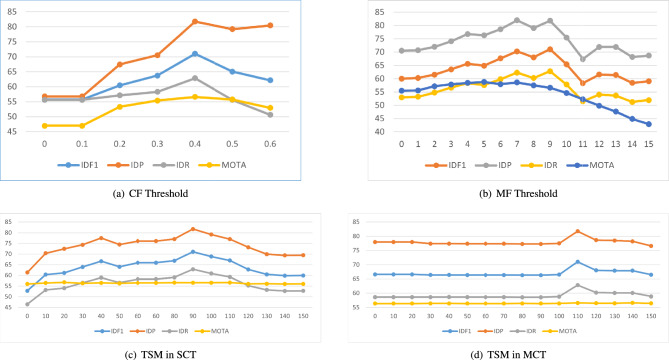
Performance Evaluation of Components in the STM. From (**a**) to (**b**), the parameters assessed are **CF**: confidence threshold of the detector filter and ** MF**: minimal displacement of matched targets Panels (**c**) and (**d**), respectively, represent the survival time of trajectories in **TSM**

To further illustrate the sensitivity of these components, we conducted a grid search to establish parameters that align with the tracking scenario. According to our experiments, as illustrated in Fig. 6, setting the Confidence Filter threshold to 0.4 ensures the quality of the detection results. The threshold for the Movement Filter is influenced by camera height, set at 9 for the HST dataset and 1 for the AICity S02. We believe this parameter is influenced by the distance between the camera and the target, thereby impacting tracking performance.

Additionally, two other manually set parameters are the buffer times for the TSM, determining when a target has exited the current camera and all cameras; these are set to 90 and 110 frames for the HST and 30 and 90 frames for the AICity S02. These parameters are related to the camera frame rate and the spatial distribution of cameras. Their design can be guided by the following control equations:

6 $$\begin{aligned} & \text {Local buffering time:}\quad \text {fps} \cdot \left( t_{\textrm{occlusion}} \cdot (1 - \eta )\right) & \end{aligned}$$

7 $$\begin{aligned} & \text {Global buffering time:}\quad \frac{D_{\textrm{cam}}}{v_{\textrm{avg}} \cdot \text {fps}} & \end{aligned}$$

where, $t_{\textrm{occlusion}}$ is the average duration of occlusion, $\eta$ is the probability of target reappearance (e.g., $\eta = 0.83$ for urban road scenarios), $D_{\textrm{cam}}$ denotes the average distance between cameras (e.g., 200 meters on highways), $v_{\textrm{avg}}$ is the average vehicle speed. These formulations provide a principled way to set buffering intervals in both local and global association processes, balancing accuracy and computational load.

**Fig. 7:**
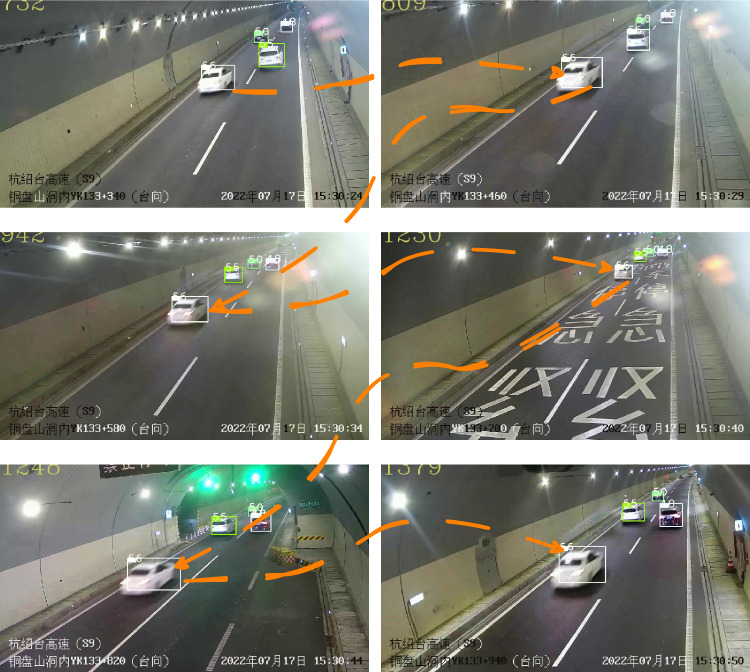
The robustness visualization of cross-camera multi-object tracking under vehicle occlusion and overtaking scenarios.

## Conclusion

Our research significantly advanced the field of MCMT tracking by developing a sophisticated online DGT framework, which operated independently of prior knowledge or delayed information. We introduced a binary graph matching approach as a superior alternative to traditional clustering methods. This new approach incorporated the iterative matching technique between cameras, enabling the MCMT system to process both live video streams and stored data effectively.

We tackled common tracking challenges, such as target loss, by devising iterative matching strategies and a Stable Trajectory Manager. To address the issue of target scale variation due to distance changes from the camera, we implemented a lightweight Hybrid Fusion Module. These enhancements have proven crucial for maintaining robust appearance representations across varying scales. From Fig. 7, it is evident that our tracker maintains stable performance in scenarios involving overtaking and interference from similar targets. Additionally, it exhibits considerable robustness to changes in scale.

Despite these advancements, our system still has limitations, which may encounter difficulties in particularly complex environments such as densely crowded areas or highly dynamic scenes. Additionally, our performance can be compromised by varying lighting conditions within specific scenes, which indicates a need for further refinements to enhance environmental adaptability.

## Data Availability

HST Dataset: The HST (Highway Surveillance Traffic) dataset was developed and annotated by our research team. Detailed information about the dataset can be found in the manuscript. If access to the dataset is required, please contact the corresponding author for further details. - AIC Dataset: The AI City Challenge S02 dataset, which utilizes the CityFlow dataset, is publicly available. It can be accessed from the official AI City Challenge website at https://www.aicitychallenge.org/2022-data-access-instructions/. Both datasets are crucial for evaluating the Dynamic Global Tracking (DGT) framework, and we encourage their use for further research with proper citation.
